# Evaluation of Six Cases of Idiopathic Gastric Antral Ulcer

**DOI:** 10.4021/gr437w

**Published:** 2012-05-20

**Authors:** Tateki Yamane, Takayuki Ishii, Akira Umeda, Hitoshi Shimao

**Affiliations:** a Division of Gastroenterology, Department Of Internal Medicine, The International University of Health and Welfare, Shioya Hospital, Japan; b Sanikukai Family Clinic, Japan; c Division of pulmonology, Department Of Internal Medicine, The International University of Health and Welfare, Shioya Hospital, Japan; d Department of Surgery, The International University of Health and Welfare, Shioya Hospital, Japan

**Keywords:** Idiopathic gastric ulcer, Gastric antral ulcer, Refractory gastric ulcer

## Abstract

Six cases of gastric antral ulcer with an unknown cause encountered at our hospital and related facilities during the last 5 years were evaluated. The frequency of the disease was 1.3% of all gastric ulcers. The lesions were multiple in 3 and solitary in 3. All these lesions were ellipsoidal and small ulcers 1 cm or less in long diameter with mucosal elevations around them, located primarily in the greater curvature, and accompanied by reddened erosions in other areas of the antrum. The patients were middle-aged or older, 5 of them were females, half of them had a history of bleeding, and 4 showed resistance to treatment with proton pump inhibitors. The 6 patients had common clinical features, suggesting that they had the same disease. From the presence of reddened erosion, mutual friction of the antral mucosa was suspected to be a cause of the disease. Similar ulcers are found in the literature, but they have not been described or evaluated in detail. The further accumulation of cases and clarification of details of the disease are desired.

## Introduction

*Helicobacter pylori* (Hp) and NSAIDs are two major causes of peptic ulcer, and Zollinger-Ellison syndrome (ZES), infections other than Hp infection, and drugs other than NSAIDs have been reported as rare causes [1]. Gastric antral ulcers are related more frequently to NSAIDs than to Hp, but they are also caused occasionally by bisphosphonate preparations and herpes simplex infection. During the 5 years between 2006 and 2010, we encountered 6 cases of idiopathic gastric antral ulcer, in which all these causes were excluded, at our hospital and related facilities. Similar cases are observed in the literature, but they have not been evaluated in detail. We report these 6 cases with a clinical review.

## Cases Report

We treated 6 patients with idiopathic gastric antral ulcer differentiated from erosion who followed a chronic course, were negative for Hp, had no history of treatment with NSAIDs, and in whom ZES, Crohn’s disease, use of drugs other than NSAIDs, and special conditions such as syphilis, tuberculosis, herpes simplex virus, and cytomegalovirus infection were excluded, at our hospital and related facilities during the 5 years between 2006 and 2010. In this study, the frequency of the condition in all cases of gastric ulcer, clinical background including the age, gender, symptoms, concurrent disorders, smoking history, drinking history, and duration of illness, endoscopic findings such as the sites of the lesions in the antrum, gross characteristics, and the presence or absence of other lesions, histopathological findings by biopsy from the lesions, serum gastrin and pepsinogen I levels, and clinical course were evaluated in these 6 cases. Hp infection was judged to be negative when the serum anti-Hp antibody was negative on condition that there was no endoscopic sign of chronic gastritis and that the results of microscopic examination and the rapid urease test were negative.

The 6 cases accounted for 1.3% of the 468 cases of gastric ulcer diagnosed and treated during the 5 years. They were aged 57 - 79 years, with a mean of 64.5 ± 18.5 years, and consisted of 5 females and 1 male. Symptoms were non-specific with epigastric pain in 4 and epigastric discomfort in 2. Hypertension was noted in 4 as a concurrent disorder. None had a smoking history, and although one had a drinking history, the patient was not a heavy drinker. The duration of illness was 0.5 - 2 years. A history of bleeding from the ulcer was present in 3 (Table 1).

**Table 1:** Clinical Characteristics

case	sex / age	complaint	complication	smoking /alcohol	history of bleeding
1	F / 68	epigastric pain	hypertension	(-)/(-)	(+)
2	F / 79	epigastric discomfort	hypertension	(-)/(-)	(+)
3	M / 57	epigastric discomfort	none	(-)/(+)	(-)
4	F / 64	epigastric pain	hypertension	(-)/(-)	(+)
5	F / 57	epigastric pain	hypertension	(-)/(-)	(-)
6	F / 62	epigastric pain	none	(-)/(-)	(-)

The ulcers were all ellipsoidal and small 10 mm or less in diameter and were accompanied by edema of the surrounding mucosa. The lesions were multiple (3-4) in 3 and single in 3, and were located primarily in the greater curvature. Reddened erosions were noted in other areas of the antrum in all cases. The histopathological findings in biopsy specimens from the peripheries of the ulcers were only erosive change of pyloric gland tissues, and no specific change was noted (Table 2).

**Table 2:** Endoscopic Findings

case	location of ulcers	number of ulcers	size of ulcers	mucosal elevation around ulcers	coexistence of reddish erosions
1	a.w. and p.w sites of g.c.	3 - 4	6 – 8 mm	(+)	(+)
2	a.w. and p.w sites of g.c.	3	6 – 8 mm	(+)	(+)
3	a.w. site of g.c.	1	7 mm	(+)	(+)
4	a.w. site of g.c.	1	7 mm	(+)	(+)
5	a.w. and p.w sites of g.c.	3	6 – 8 mm	(+)	(+)
6	a.w. site of g.c.	1	7 mm	(+)	(+)

a.w.: anterior wall, p.w.: posterior wall, g.c.: greater curvature

The serum gastrin level was measured in all cases and was in the normal range, being 124 - 170 pg/mL. The pepsinogen I level, measured in 4, was 78.8 - 97.8 ng/mL, showing no abnormality (Table 3).

**Table 3:** Laboratory Findings

case	serum gastrin (pg/mL)	pepsinogen I (ng/mL)
1	156	not measured
2	128	not measured
3	124	97.8
4	170	78.8
5	136	89.7
6	154	92.3

All cases were treated with a proton pump inhibitor (PPI) labeprazole (LPZ) at 30 mg/day. Two showed scarring after 8 weeks, but the other 4 resisted treatments and were not cured even after 12 weeks. Since nocturnal gastric acid breakthrough (NAB) was noted on 24-hour intragastric pH monitoring in 1 of these 4 cases, LPZ at 15 mg in the morning was combined with the H2-receptor antagonist (H2-RA) ranitidine (RAN) at 150 mg before sleep, resulting in scarring, pH monitoring was not performed in the other 3 refractory cases, because they did not consent to the examination, but they were also tentatively treated with the above combination of PPI and H2-RA, which was effective in 1, resulting in scarring, but ineffective in 2 (Table 4).

**Table 4:** Reaction to Medication

case	PPI	combination of PPI and H2-RA
1	not healed	healed
2	healed	-
3	not healed	not healed
4	healed	-
5	not healed	not healed
6	not healed	healed

The clinical courses, endoscopic findings, and biopsy-based histopathological findings of 3 typical cases are presented.

### Case 1

**Figure 1:**
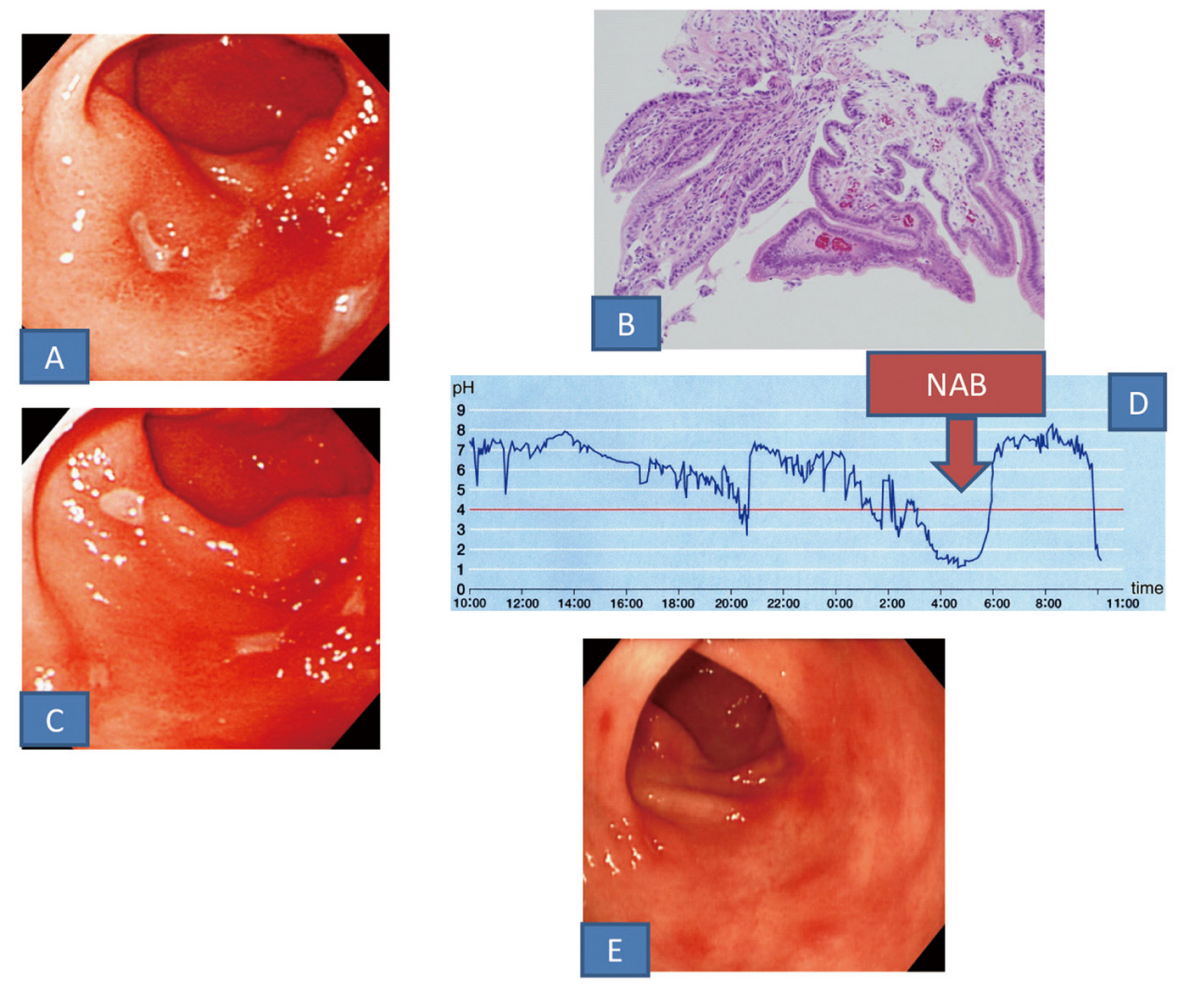
A: Upper gastrointestinal endoscopic findings. Small ulcers with edema in the surrounding mucosa were noted in the greater curvature of the antrum. B: Histopathological findings of a biopsy specimen (HE staining). Eroded pyloric tissue with no specific features was seen. C: Endoscopic findings. After administration of PPI the ulcers did not healed. D: 24-hour intragastric pH monitoring finding. NAB was found (arrow). E: Endoscopic findings. After administration of PPI and H2-RA in combination the ulcers scared.

This 68-year-old woman had a history of hypertension and was being treated orally with an antihypertensive agent (angiotensin II receptor blocker). Two years before, she was diagnosed with an antral ulcer and was treated at another hospital, but was referred to our department due to refractoriness of the disease. The primary symptom was epigastric pain, and there was a history of bleeding from the ulcer. The serum gastrin level was 156 pg/mL, and no abnormality was noted on other blood tests. The fecal occult blood test performed for the differential diagnosis from diseases including Crohn’s disease (immunological method) was also negative. On the first endoscopy at our hospital, 3 small ulcers with edema in the surrounding mucosa were noted near the anterior and posterior walls of the antral greater curvature, and reddened erosions were also observed in other areas of the antral greater and lesser curvatures (Fig. 1A). The biopsy-based histopathological findings from the peripheries of the ulcers were eroded pyloric gland tissue showing no significant change (Fig. 1B). The H2-RA(RAN, 300 mg/day) prescribed by the previous doctor was substituted for LPZ at 30 mg/day, but the ulcers were not cured at endoscopy after 8 weeks, and the number of ulcers was increased to 4 after 12 weeks (Fig. 1C). Since NAB was noted by 24-hour intragastric pH monitoring (Fig. 1D), 15 mg LPZ in the morning was coupled with 150 mg RAN before sleep, and scarring of the ulcers was confirmed by endoscopy after 4 weeks (Fig. 1E). This regimen is still being continued.

### Case 3

**Figure 2:**
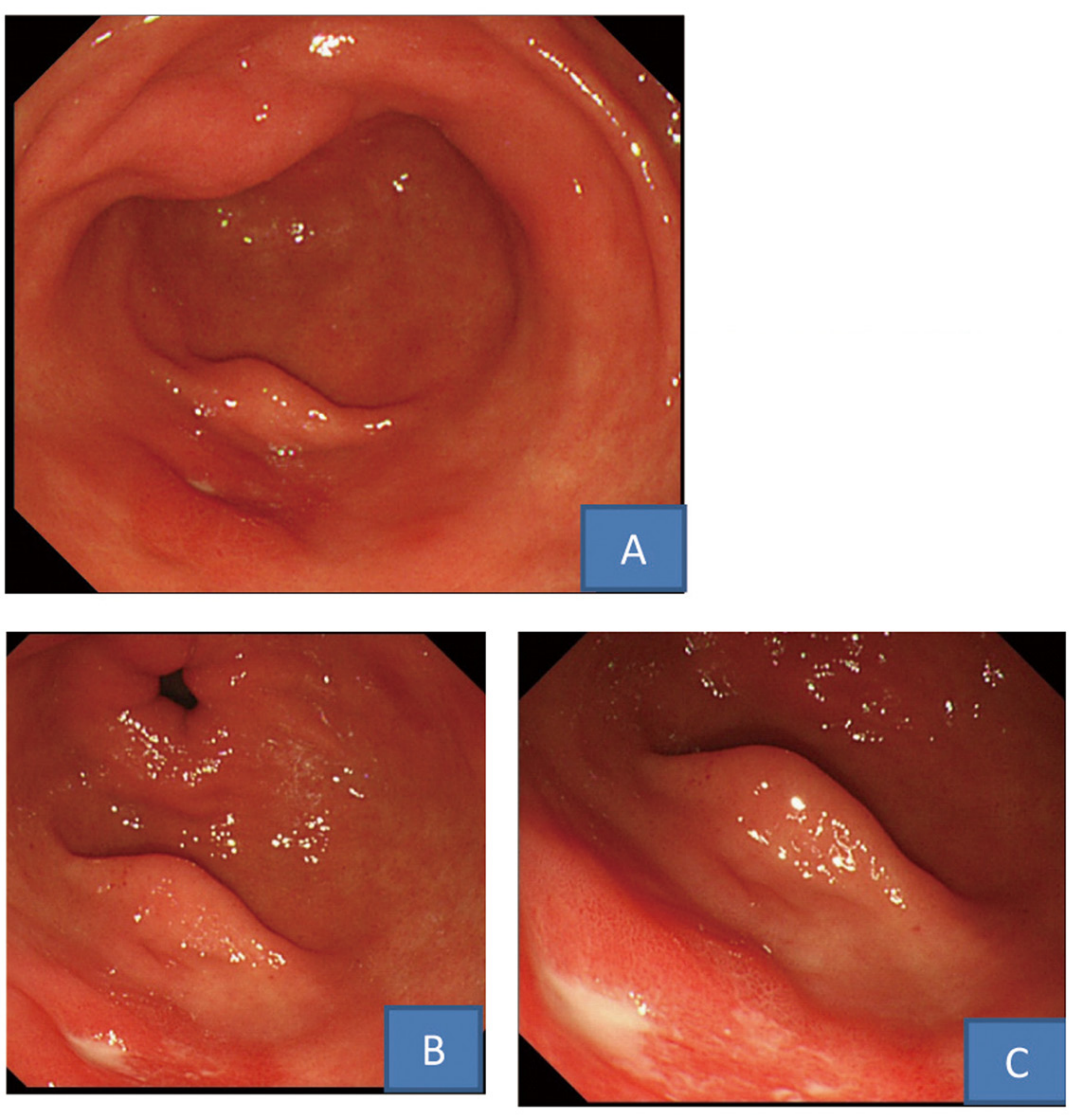
Upper gastrointestinal endoscopic findings. A: A small ulcer with edema in the surrounding mucosa was found in the greater curvature of the antrum. B: After administration of PPI the ulcer did not healed. C: After administration of PPI and H2-RA in combination the ulcer was not cured.

This 57-year-old man with no particular history consulted our department due to epigastric discomfort, which had persisted for 6 months. Endoscopy revealed a small ulcer accompanied by edema of the surrounding mucosa, and smoothly elevated reddened erosion was observed in other areas of the antral greater and lesser curvatures (Fig. 2A). Biopsy specimens from the ulcers yielded no significant finding. The serum gastrin and pepsinogen I levels were normal at 124 pg/mL and 97.8 ng/mL, respectively, no abnormality was noted in the other blood test results, and the fecal occult blood test (immunological method) was negative. The ulcer showed no change even 12 weeks after the beginning of the administration of LPZ at 30 mg/day (Fig. 2B). He did not consent to 24-hour intragastric pH monitoring. Although he was administered PPI and H2-RA in combination (15 mg LPZ in the morning + 150 mg RAN before sleep) similarly to Case 1, the ulcer was not cured (Fig. 2C). Thereafter, RPZ was administered with protection factor potentiators such as prostaglandins, but cure of the ulcer could not be achieved.

### Case 6

**Figure 3:**
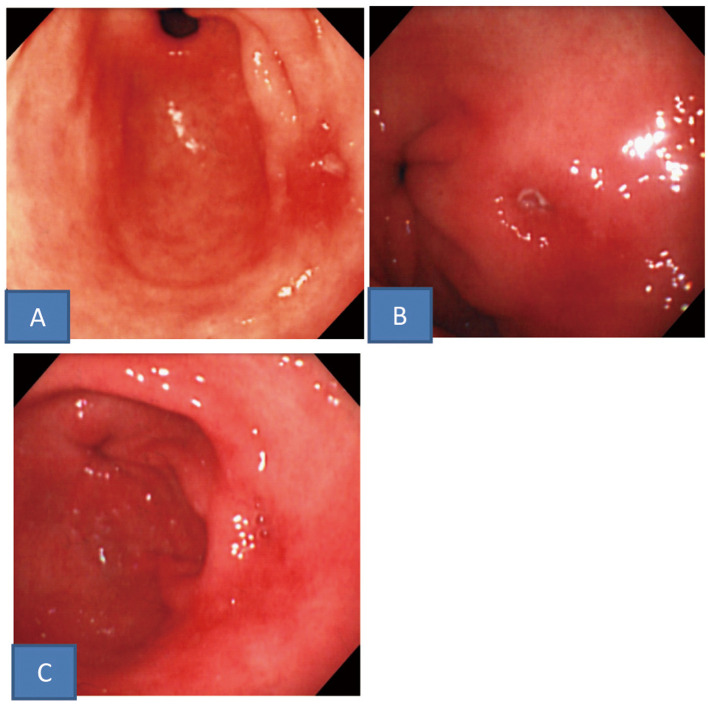
Upper gastrointestinal endoscopic findings. A: A small ulcer with edema in the surrounding mucosa was noted in the greater curvature of the antrum. B: After administration of PPI the ulcer did not healed. C: After administration of PPI and H2-RA in combination the ulcer scared.

This 62-year-old woman with no particular history consulted our department half a year before due to epigastric pain. On endoscopy, a small ulcer accompanied by edema of the surrounding mucosa was noted near the posterior wall of the antral greater curvature with reddened erosion in other areas of the antrum (Fig. 3A). No significant finding was obtained by histopathological examination of biopsy specimens from the ulcer. The serum gastrin and pepsinogen I levels were normal at 154 pg/mL and 92.3 ng/mL, respectively, and no abnormality was noted on the other blood tests or fecal occult blood test (immunological method). No cure of the ulcer was observed even after the 12-week administration of LPZ at 30 mg/day (Fig. 3B). She did not consent to 24-hour intragastric pH monitoring, but PPI and H2-RA were administered in combination (15 mg LPZ in the morning + 150 mg RAN before sleep), and endoscopy performed after 4 weeks showed scarring of the ulcer (Fig. 3C). She has been observed to date while continuing the same regimen.

## Discussion

Hp infection and NSAIDs are frequent causes of peptic ulcer, and the disease is also caused rarely by ZES, infections including cytomegalovirus infection, herpes simplex virus infection, tuberculosis, syphilis, drugs other than NSAIDs including bisphosphonate, Crohn’s disease, ulcerative colitis, and circulation disorders of the mucosa due to portal hypertension or atherosclerotic diseases [1], but idiopathic ulcer without a clear cause is also observed. The incidence of non-Hp, non-NSAIDs gastric ulcers including idiopathic ulcer is relatively high at 10-20% in Western countries [2, 3], but is low at less than 10% in Asia [4] and has recently been reported in Japan to be 2% [1]. Idiopathic cases have been suggested to be related to smoking, psychological stress, and *Helicobacter heilmanii* infection, but there is no consensus. On the other hand, gastric antral ulcers are caused rarely by Hp and often by NSAIDs, but Alendronate, a bisphosphonate preparation, is also known to cause multiple small ulcers in the antrum. These drugs are considered to cause antral ulcers by direct mucosal damage due to concentration of the drugs during ejection of the gastric contents. Herpes simplex virus infection has also been suggested as a rare cause of multiple small ulcers of the antrum. The 6 patients evaluated in this study were negative for Hp and had no history of the use of NSAIDs or bisphosphonate, and showed no symptom or inflammatory reaction suggestive of herpes virus infection. ZES and other infection and Crohn’s disease were also excluded by laboratory findings including the serum gastrin level and clinical symptoms. The pepsinogen I level was normal in the 4 patients in whom it was measured, and hyperacidity was considered unlikely. Thus, the 6 patients were diagnosed with idiopathic antral ulcer with an unknown cause. An elevated erosion covered by fur is occasionally noted in the antrum of non-Hp-infected individuals [5], but the lesions in our 6 patients were clearly ulcers rather than erosive changes.

The ulcers in the 6 patients were multiple and solitary in 3 each, but they were all small ulcers located primarily in the greater curvature accompanied by edema of the surrounding mucosa and reddened erosions in other areas of the antrum. They also exhibited many common endoscopic features. The patients were mostly middle-aged or older women and showed common clinical features including resistance to PPIs, suggesting that they were in the same pathological condition.

Since reddened erosions were observed in other parts of the antrum in all 6 patients, mutual friction of the antral mucosa due to excessive peristalsis was considered to be a cause of the ulcers although objective examinations such as intragastric pressure measurement and electrogastrography were not performed. However, as cure was observed by the administration of gastric acid secretion inhibitors in 4 but not in 2, an involvement of gastric acid was considered possible similarly to usual peptic ulcer.

The disease resisted PPI and was refractory in 4 of the 6 patients. For the establishment of a peptic ulcer, dehydration and fixation of the tissue by gastric acid are necessary, and ulcers are usually cured by controlling the gastric acid secretion with PPI or H2-RA. However, 2.5-4% of the gastric ulcers have been reported to resist PPI, a potent acid secretion inhibitor [6], and a large size, deep undermining , an irregular shape, and circumferential elevation have been reported as their morphological characteristics [7]. Ulcers with such morphological features have been reported to have factors of refractoriness such as the histopathological growth of callous collagen fibers in the bottom, which prevents shrinking of the ulcer, circulatory disturbance, which prevents the appearance of regeneration epithelium, and mucosal fibrosis, which interferes with epithelial maturation [8]. Although the ulcers observed in our patients were small, the edema of the mucosa around them is considered to correspond to the circumferential elevation in the above report, and cure is considered to have been difficult to achieve due to factors including callus formation. Also, resistance to acid secretion inhibitors may have been due to the mechanical friction mentioned above. The resistance to PPI may have been caused by insufficiency of its activity because of inactivation in the stomach due to disorder of ejection into the duodenum or acceleration of its metabolism due to gene polymorphism [9, 10]. However, in the 4 PPI-resistant patients, no deformity of the stomach such as wallet stomach was noted; endoscopy showed no food retention in the stomach, and ejection disorder of PPI into the duodenum was excluded. Also, acceleration of metabolism as in rapid metabolizers is not considered to occur on the long-term administration of PPI, unlike short-term administration for the eradicatin of Hp. For these reasons, although we did not perform intragastric pH monitoring, insufficiency of the activity of PPI is considered unlikely in the 4 patients.

In 1 of the 4 patents, 24-hour intragastic pH monitoring revealed NAB, and the administration of PPI and H2-RA in combination was effective. In the other 3 patients, pH monitoring was not performed, but the combined administration was effective in 1 of them. NAB due to the insufficient inhibition of gastric acid secretion by PPI during the night-time has been reported to be a cause of severe reflux esophagitis and to be observed frequently in Hp-negative patients. For its treatment, the concomitant administration of H2-RA, which has an excellent gastric acid secretion inhibitor activity, and an increase in the dose and fractionated administration of PPI are recommended [11, 12]. There is an opinion that the inhibition of nocturnal acid secretion may be effective for the treatment of severe reflux esophagitis but not for the treatment of common gastric ulcers. However, as there has been no evaluation regarding NAB in patients with refractory peptic ulcer, the responses to the combination of PPI and H2-RA observed in our 2 patients are interesting. The continuous administration of H2-RA has been reported to cause tolerance and attenuation of the acid secretion inhibitor effect in Hp-negative patients, but there is also a report that its inhibitory effect on nocturnal acid secretion is maintained [13]. We intend to follow-up these 2 patients and see whether cure of the ulcer can be maintained by the continuous administration of H2-RA with PPI. In the other 2 refractory cases, the treatment remains difficult as no scarring has been achieved despite the administration of PPI in combination with mucosal protection factor potentiators such as prostaglandins, and we are evaluating stronger acid secretion inhibitor therapies such as doubling the dose of PPI.

By searching the *Japana Centra Revuo Medicina* and MEDLINE for the literature published between 1990 and 2010 using “non-Hp, non-NSAID ulcer”, “idiopathic peptic ulcer”, and “gastric antral ulcer” as key words, we found 1 case of idiopathic gastric antral ulcer reported by Tsuji et al [14], 2 cases reported by Nishikawa et al [1], and 2 cases reported by Aoyama et al [15] in their papers concerning peptic ulcer. While no detailed information concerning the morphology or clinical course was available, these reports suggest that idiopathic gastric antral ulcers have been reported sporadically. We hope that cases are accumulated further and that the etiology of this ulcer disease is clarified in the future.

### Conclusion

Six cases of idiopathic gastric antral ulcer were reviewed. They showed common clinical features and were suspected to have the same disease.
